# F22. DYSLIPIDEMIA AND INFLAMMATORY MARKERS IN RELATION TO CLINICAL SYMPTOMATOLOGY IN PSYCHOTIC DISORDERS

**DOI:** 10.1093/schbul/sby017.553

**Published:** 2018-04-01

**Authors:** Sherif M Gohar, Ingrid Dieset, Nils Eiel Steen, Ragni H Mørch, Trude S Iversen, Vidar M Steen, Ole A Andreassen, Ingrid Melle

**Affiliations:** 1 NORMENT K.G. Jebsen Centre for Psychosis Research, Oslo University Hospital, Institute of Clinical Medicine, University of Oslo; 2 NORMENT K.G. Jebsen Center for Psychosis ResearchUniversity of Bergen

## Abstract

**Background:**

Although the role of lipid disturbances and inflammation in psychotic disorders have been demonstrated in a substantial body of research, the association between clinical symptomatology and these two important pathways has not been studied in detail. The main aim of this study was to investigate the associations between serum lipid levels [total cholesterol (TC), low density lipoprotein (LDL), triglyceride (TG)]; general or specific inflammatory markers [C-reactive protein (CRP), soluble tumor necrosis factor receptor 1(sTNF-R1), osteoprotegerin (OPG), interleukin 1 receptor antagonist (IL-1Ra)]; and clinical symptoms (positive, negative and depressive) in patients with psychotic disorders.

**Methods:**

The sample is consisted of 652 participants divided in two groups: Schizophrenia, schizophreniform and schizoaffective, (schizophrenia group, N = 344); psychosis NOS, psychotic bipolar I, II and NOS, (non-schizophrenia group, N = 308) recruited consecutively between 2003 and 2015 from five major hospitals in Oslo, Norway, as part of Thematically Organized Psychosis (TOP) Study. The Regional Committee for Medical Research Ethics approved the study. Demographic, clinical and medications data were obtained by clinical interviews and from medical records. SCID-I was used for diagnosis in addition to Positive and Negative Syndrome Scale (PANSS) and Calgary Depression Scale for Schizophrenia (CDSS) to assess symptoms severity. Bivariate and multivariate analyses were performed to evaluate associations between symptom profiles, lipid levels and inflammatory markers.

**Results:**

Schizophrenia group showed higher levels of TC and LDL compared to non-schizophrenia group after adjusting for age, gender, BMI, smoking, and medications. TC and LDL were positively correlated with depression, whereas TG and LDL were positively correlated with negative symptoms. CRP and OPG were significantly associated with higher levels of TC and LDL. While, sTNF-R1 showed significant positive correlation only with TG. In multivariate regression, higher LDL was significantly associated with higher age, BMI, depressive severity in addition to two inflammatory markers CRP and OPG.

**Discussion:**

The findings of this study highlight the importance of understanding the interaction between inflammatory markers and lipid, and their relation to clinical profile especially depression in psychotic disorders

